# Development of a Bionic Bistable Compliant Mechanism for the LDI Machine

**DOI:** 10.3390/mi17060640

**Published:** 2026-05-22

**Authors:** Ruizhou Wang, Junhong Li, Hua Wang

**Affiliations:** 1 State Key Laboratory of Precision Electronic Manufacturing Technology and Equipment, Guangdong University of Technology, Guangzhou 510006, China; junhongli123@163.com; 2 Guangdong Provincial Key Laboratory of Industrial Intelligent Inspection Technology, Foshan University, Foshan 528000, China; 3 Guangdong Provincial Engineering Technology Research Center of Digital Lithography, Guangdong KST Optical Co., Ltd., Dongguan 523000, China; kstuv@kstuv.com

**Keywords:** compliant mechanism, bistable mechanism, bionic mechanism, laser direct imaging (LDI)

## Abstract

Rigid mechanisms (RMs) are widely adopted in the vision-based measurement (VBM) system of laser direct imaging (LDI) machines. Constant-stiffness compliant mechanisms (CMs) improve the performance of traditional RMs. Unfortunately, constant-stiffness CMs still exhibit high energy consumption and limited adaptability during fast focusing. Inspired by the hierarchical structure and mechanical behavior of ligaments and tendons, this paper proposes a bionic bistable compliant mechanism (BBCM) to replace constant-stiffness CMs. The BBCM exhibits dynamic stiffness characteristics throughout the focusing stroke, with low stiffness in the transition phase to reduce energy consumption during rapid focusing and high local stiffness near the stable positions to maintain focusing stability. A numerical model is established to analyze the variable-stiffness and bistable characteristics of the proposed BBCM. Prototype tests demonstrate the bistable response, dynamic feasibility, and energy-saving potential of the mechanism. Under the tested camera-loaded flying-shot condition, compared with the constant-stiffness CM, the BBCM reduces electrical and mechanical energy consumption by 12.37% and 9.74%, respectively. The target recognition results indicate that the BBCM-based system maintains comparable visual measurement performance. These results demonstrate that the proposed BBCM provides a feasible mechanism-level solution for energy-efficient dual-position focusing in LDI machines.

## 1. Introduction

Digital lithography is a critical process in the manufacturing of chips and integrated circuits (ICs) [1,2]. Laser direct imaging (LDI) lithography offers advantages such as high precision, maskless operation, and low cost [3,4]. It is widely used for exposing circuit patterns on high-density interconnect (HDI) IC substrates and printed circuit boards (PCBs) [5]. LDI machines use vision-based measurement (VBM) systems to establish a mapping relationship between the substrate and the digital mask, ensuring exposure accuracy [6,7]. Unfortunately, the measurement accuracy of VBM systems is affected by the quality of the captured target images. To address this issue, the VBM system must adjust the camera focus during target image acquisition.

Flying-shot technology captures images during continuous motion and improves measurement efficiency [8]. In recent years, it has been applied to VBM systems in LDI machines [9]. Flying-shot focusing is critical to ensuring the measurement accuracy of LDI machine VBM systems [10]. High-speed flying-shot focusing requires the camera module to achieve rapid, accurate, and stable focal-plane adjustment [11]. However, existing focusing mechanisms still face limitations under high-frequency focal-plane switching conditions. Conventional rigid mechanisms can provide a relatively large stroke, but their performance is limited by friction, clearance, assembly errors, and vibration induced by frequent start–stop or reverse motion. Constant-stiffness compliant mechanisms avoid friction and clearance through elastic deformation, but they maintain nearly constant elastic resistance over the whole focusing stroke. Therefore, frequent focal-plane switching requires continuous driving force and leads to increased energy consumption. These limitations make it difficult for existing mature schemes to simultaneously satisfy rapid switching, imaging stability, and low-energy focusing in LDI flying-shot VBM systems.

Bistable mechanisms are mechanical structures that can switch between two stable states and self-lock in either state without external force [12]. Bistable compliant mechanisms utilize the elastic deformation of hinges to form two stable positions within the motion range, simultaneously combining bistable characteristics with high motion precision [13]. Recent studies have further extended bistable compliant mechanisms from basic switching structures to application-oriented compliant systems. For example, hair-clip-inspired bistable mechanisms have been used to enhance the rapid undulating motion of soft robotic fish [14], while typical bistable beams have been introduced as negative-stiffness elements to achieve adaptive constant-force output in compliant soft robotic fingers [15]. These studies indicate that bistable compliant mechanisms have shown potential in rapid actuation, energy storage and release, and stiffness regulation. However, most existing bistable designs are mainly developed for switching, locking, actuation, or force-regulation tasks, and they do not directly address the coupled requirements of small-stroke focal-plane switching, high local stiffness at imaging positions, and low-energy transition in LDI flying-shot VBM systems. If the steady-state positions of the bistable compliant mechanism are designed as the focal planes, the system operates in a high-stiffness state during image acquisition [16]. Meanwhile, during the transition between the two steady-state positions, the system operates in a variable-stiffness state [17]. Based on this feature, the proposed BBCM is developed to match the two focal planes of the LDI flying-shot process. It provides high local stiffness near the two imaging positions to suppress vibration-induced defocusing, while reducing the equivalent stiffness during focal-plane transition to decrease the required driving force and energy consumption.

Multi-focus (MF) image fusion technology combines images captured at multiple focal planes to produce clear images for visual measurement. With the rapid advancement of image processing technology, image fusion techniques can be categorized into transform-domain image fusion, spatial-domain image fusion, and deep learning-based image fusion methods [18,19]. These techniques provide the technical foundation for high-precision visual measurement in the VBM system of LDI machines. When exposing PCB circuit patterns, circular targets are typically used as visual recognition targets. The center coordinates and radius are key information for target recognition [20]. The Random Hough Transform (RHT) algorithm is commonly used for circular target recognition due to its high accuracy and efficiency. However, the RHT algorithm suffers from poor robustness [21]. Image blurring, variations in exposure intensity, and abnormal shapes may cause the algorithm to fail [22]. Therefore, it is necessary to develop a more robust target recognition algorithm for the VBM system of LDI machines.

To address the above issues, this paper proposes a voice coil motor (VCM)-driven bionic bistable compliant mechanism (BBCM), inspired by the structure of finger ligaments [23,24,25]. The BBCM is used for rapid focal-plane switching in the flying-shot VBM system of an LDI machine [26]. A theoretical model of the BBCM is established based on beam constraint theory, and its stiffness characteristics, bistable behavior, and focusing energy consumption are analyzed. In addition, a guided-filter-based multi-focus image fusion method is introduced, and an RRHT-based target recognition method is adopted to extract the center coordinates and radius of circular targets. Finally, a prototype system is built to test the bistable characteristics and energy consumption of the BBCM, and an LDI experimental platform is established to evaluate the in-service performance of the BBCM, the multi-focus image fusion method, and the RRHT-based target recognition method.

## 2. Mechanism Design of BBCM Inspired by the Ligament

According to the installation and focusing requirements of laser direct imaging equipment, the proposed mechanism should satisfy several constraints. First, the overall structure should be sufficiently compact so that it can be installed within a 165 mm × 200 mm installation space without interfering with the original motion chain. Second, substrate warpage and external environmental vibration may cause a defocusing displacement of up to approximately 1.00 mm. Since the depth of focus of the selected optical lens is approximately 0.54 mm, the effective focusing stroke of the mechanism is designed to be approximately 2.00 mm, which is nearly four times the depth of focus. This stroke setting enlarges the axial focusing coverage and satisfies the requirement of dual-focal-plane acquisition. Third, the mechanism should be able to carry the camera, lens, and light-source assembly, with a rated working load of approximately 336 g. In addition, the mechanism should maintain high local stiffness near the two imaging positions to improve focusing stability while reducing the equivalent stiffness in the transition region to decrease the driving force and energy consumption during frequent focal-plane switching. Based on the above requirements, a bionic bistable compliant mechanism inspired by the nonlinear stiffness characteristics of ligaments is designed in the following section.

### 2.1. Concept Design of Bionic Inspiration

**Figure 1:**
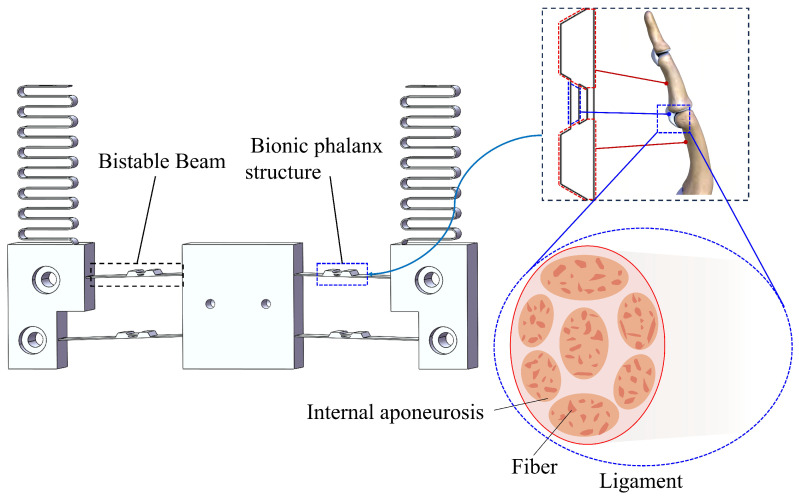
Design of BBCM inspired by the ligament.

Ligaments exhibit nonlinear mechanical properties characterized by a J-shaped load-displacement curve. This curve reflects their distinct biomechanical behavior, where a low-stiffness toe region transitions into a high-stiffness linear region. The bionic design of the BBCM is inspired by the hierarchical structure and unique mechanical behavior of ligaments and tendons in biological systems, as shown in Figure 1.

In the relaxed state, the ligament fibers exhibit a wavy, folded configuration. When subjected to tensile forces, these fibers gradually straighten, resulting in increased stiffness. By incorporating a ligament-inspired bionic structure, the mechanism provides low stiffness in the relaxed state and high stiffness in the tensioned state, thereby achieving dynamic stiffness adjustment.

In practical application, due to the size constraints of the VCM used for focusing, the motor may occasionally lack sufficient driving force during rapid focusing motions. To address this, a set of corrugated spring sheets is introduced into the mechanism. These spring sheets act as an auxiliary elastic element, storing and releasing additional energy during the transition between stable states, thereby compensating for potential drive deficits and ensuring focusing performance.

### 2.2. Concept Design of Bistable Configuration

The BBCM is designed based on the nonlinear mechanical characteristics of biological ligaments [27]. Biological ligaments generally exhibit a nonlinear tensile response, in which the equivalent stiffness is relatively low under small deformation and increases as the elongation becomes larger. To describe this ligament-inspired nonlinear stiffening trend in a dimensionally consistent form, an empirical normalized force–displacement relationship is introduced as

(1) $$F_{b}=F_{0}{\left(\frac{\Delta L}{L_{0}}\right)}^{n}$$

where $F_{b}$ is the equivalent tensile force exerted by the ligament-like structure, $\Delta L=L-L_{0}$ is the elongation, $L_{0}$ is the initial characteristic length, $F_{0}$ is an empirical force coefficient with the unit of N, and *n* is a dimensionless nonlinear exponent. n>1 is used to represent the nonlinear stiffening tendency inspired by biological ligaments. The elastic potential energy associated with this simplified nonlinear tensile response is obtained by integrating the force–displacement relationship as

(2) $$U_{b}=\int_{0}^{\Delta L}F_{b}\left(s\right)\;ds=\frac{F_{0}L_{0}}{n+1}{\left(\frac{\Delta L}{L_{0}}\right)}^{n+1}$$

where *s* is the integration variable and $U_{b}$ denotes the elastic potential energy of the simplified ligament-like element. It should be noted that the above empirical relationship is used only to describe the ligament-inspired nonlinear stiffening tendency, rather than to directly predict the complete bistable response of the BBCM. The actual bistability of the BBCM is determined by the geometric nonlinearity and snap-through deformation of the compliant beams, which are further analyzed using the BCM and finite element simulation in the following sections.

**Figure 2:**
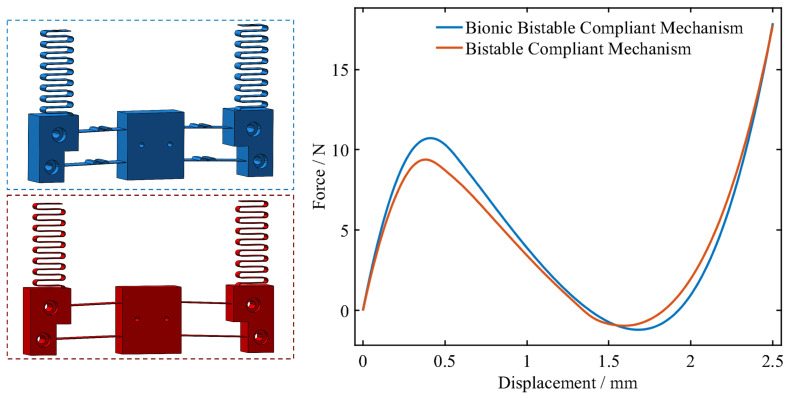
Comparison of the stiffness curves between bionic structures and non-bionic structures.

The ligament-like structure mimics the mechanical properties of biological ligaments and enables dynamic stiffness regulation. Compared with the non-bionic bistable compliant mechanism, the BBCM introduces a phalanx-like structure on the compliant beams. This phalanx-like structure changes the force transmission path and achieves a ligament-inspired nonlinear stiffness regulation effect during deformation. The effect of the bionic design is evaluated, as shown in Figure 2. The force–displacement curves of the bionic and non-bionic structures are also compared.

Both curves exhibit a positive force peak, a negative force valley, and a second zero-force equilibrium point, confirming their bistable behavior. For the bionic structure, the positive peak force is 10.72 N at 0.4 mm, and the negative peak force is −1.18 N at 1.7 mm. For the non-bionic structure, the corresponding values are 9.37 N at 0.4 mm, and −0.94 N at 1.6 mm. The peak-force difference $\Delta F=F_{\max}-F_{\min}$ increases from 10.31 N to 11.90 N after introducing the bionic unit, corresponding to an increase of 15.42%. This result shows that the bionic design strengthens the bistable effect and improves the stiffness response of the mechanism.

### 2.3. Design of a Flying Focusing VBM System Using the BBCM

The core component of the flying focusing VBM system is the BBCM. The mechanism is connected to the base plate through a transition fit with positioning pins, which helps avoid accuracy loss caused by hinge deformation during assembly.

From the kinematic viewpoint, the slider of the voice coil motor drives the shuttle of the BBCM to move along the optical axis of the lens module. The axial displacement generated by the BBCM is transmitted to the lens and camera module through the output displacement connecting plate. The force transmission path starts from the VCM, passes through the BBCM and the output connecting plate, and finally acts on the optical module. During focal-plane switching, the VCM first provides the driving thrust to overcome the nonlinear restoring force of the BBCM and the inertial load of the moving optical module.

**Figure 3:**
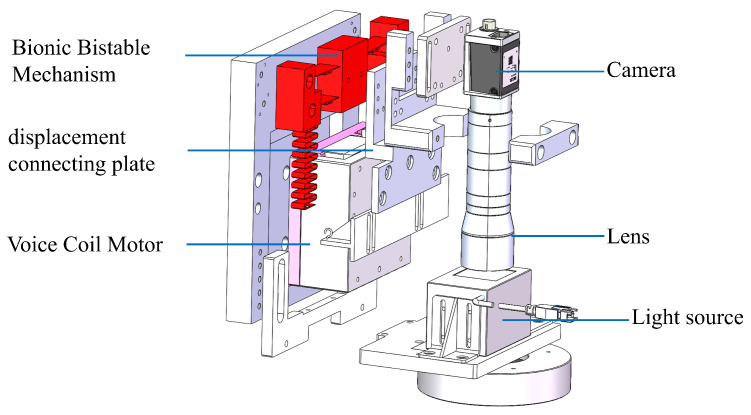
Design of a flying focusing VBM system using the proposed BBCM (marked in red).

Then, the BBCM converts the input thrust into axial focusing displacement through elastic deformation and bistable switching. The selected VCM is the XRV115 voice coil motor by Akribis, which can provide a continuous thrust of 26.32 N and a peak thrust of 79.0 N. Therefore, it is sufficient to overcome the approximately 10 N internal restoring force of the mechanism together with the 1 kg load, and it can drive the camera-loaded optical module during rapid switching. In terms of timing compatibility, the field of view of the selected lens is approximately 8 mm × 8 mm. At a flying-shot velocity of 50 mm/s, the available imaging time window is approximately 160 ms. The maximum velocity and acceleration of the VCM are 300 mm/s and 30,000 mm/s^2^, respectively, and the theoretical time required for a 2 mm focusing stroke is approximately 16.33 ms. Therefore, the focal-plane switching can be completed within the available flying-shot imaging time window, as shown in Figure 3.

**Figure 4:**
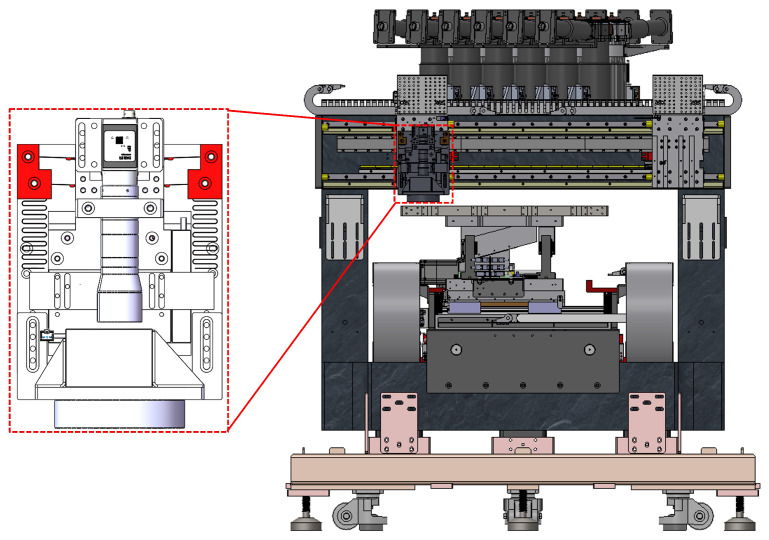
Integration of the BBCM-based VBM system inside the LDI machine.

To further enhance measurement accuracy and dynamic performance, an LDI machine was designed with a flying focusing VBM system based on a bistable mechanism. This system utilizes the collaboration between a VCM and a bistable compliant mechanism to provide support for high-precision measurements in dynamic environments. The hardware design is shown in Figure 4.

The flying focusing VBM system is integrated into the core framework of the LDI machine. The VBM system uses a high-rigidity marble base as a stable foundation. The BBCM utilizes bistable characteristics to ensure low-energy and high-precision focusing in a complex environment.

## 3. Analysis of the BBCM

The proposed BBCM is used to generate axial focusing motion along the optical axis of the lens module. During the analysis, the input displacement is applied to the shuttle of the bistable compliant mechanism along the focusing direction, and the corresponding force, stiffness variation, and bistable equilibrium positions are solved within the prescribed stroke range.

**Figure 5:**
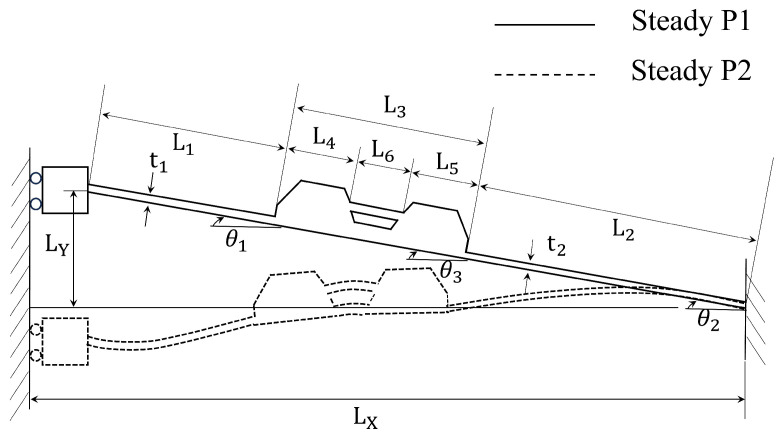
Schematic showing the parameterization of the BBCM. The out-of-plane thickness of the mechanism is denoted by *w*.

Considering that the geometry and loading of the BBCM are symmetric with respect to its vertical centerline, a single bistable fixed-guided segment is analyzed, as illustrated in Figure 5.

A bistable fixed-guided segment consists of four flexible beam elements and one rigid connecting segment. The key geometric parameters are also presented in Figure 5. The subscripts 1 and 2 correspond to flexible beam elements 1 and 2, respectively.

The out-of-plane thickness of the mechanism is denoted by *w*. The lengths of the two flexible beams are represented by $L_{1}$ and $L_{2}$, while the length of the rigid segment is denoted by $L_{3}$. The corresponding flexural rigidities of the beams are given as $E_{1}I_{1}$ and $E_{2}I_{2}$. The bistable segment is fixed at one end and connected to the shuttle of the BBCM at the other end. To accurately model the behavior of these beam elements, we apply the beam constraint model (BCM) [28,29]. The constraints and deformations of flexible beams are calculated.

### 3.1. Quasi-Static Force–Displacement Model of the BBCM Using the BCM Approach

**Figure 6:**
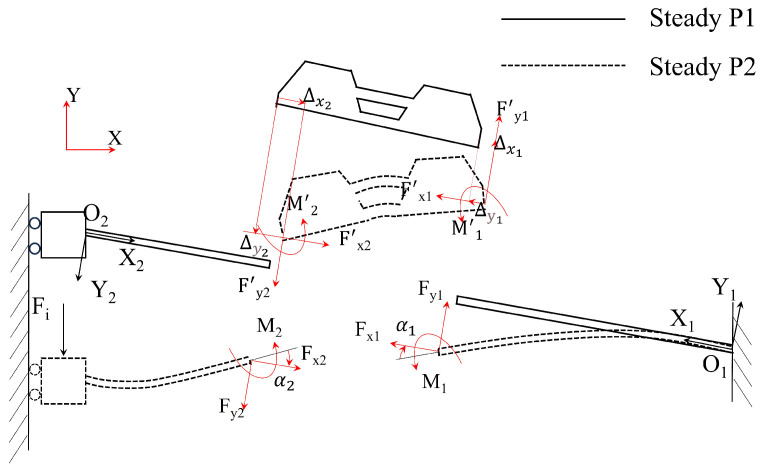
Free-body diagram of the BBCM, showing the positive directions of forces and deflections.

For the convenience of modeling and analysis, the origins of the local coordinate systems are defined at the fixed ends of Beam 1 and Beam 2 as $O_{1}\left(X_{1}Y_{1}\right)$ and $O_{2}\left(X_{2}Y_{2}\right)$, respectively, following the quasi-static modeling framework for compliant bistable mechanisms [30]. The tip deflections of each beam under loading include horizontal displacement Δx, vertical displacement Δy, and angular change α. Specifically, the tip deflections of Beam 1 are $\Delta x_{1}$, $\Delta y_{1}$, and $\alpha_{1}$, while those of Beam 2 are $\Delta x_{2}$, $\Delta y_{2}$, and $\alpha_{2}$. In addition, the external loads acting on the model include horizontal forces $F_{x1}$ and $F_{x2}$, vertical forces $F_{y1}$ and $F_{y2}$, as well as moments $M_{1}$ and $M_{2}$. Owing to the symmetry of the mechanism, only half of the structure is modeled. The corresponding free-body diagram and the positive directions of forces and deflections are shown in Figure 6.

The deflection parameters and the load parameters can be non-dimensionalized as:

(3) $$\begin{array}{cc} m_{1} & =\frac{M_{1}L_{1}}{E_{1}I_{1}},\;f_{y1}=\frac{F_{y1}L_{1}^{2}}{E_{1}I_{1}},\;f_{x1}=\frac{F_{x1}L_{1}^{2}}{E_{1}I_{1}},\;\delta_{y1}=\frac{\Delta_{y1}}{L_{1}},\;\delta_{x1}=\frac{\Delta_{x1}}{L_{1}}\\ m_{2} & =\frac{M_{2}L_{2}}{E_{2}I_{2}},\;f_{y2}=\frac{F_{y2}L_{2}^{2}}{E_{2}I_{2}},\;f_{x2}=\frac{F_{x2}L_{2}^{2}}{E_{2}I_{2}},\;\delta_{y2}=\frac{\Delta_{y2}}{L_{2}},\;\delta_{x2}=\frac{\Delta_{x2}}{L_{2}}. \end{array}$$

Applying the BCM to both beam flexures yields

(4) $$\left[\begin{array}{c} f_{y1}\\ m_{1} \end{array}\right]=\left[\begin{array}{cc} g_{11} & g_{12}\\ g_{21} & g_{22} \end{array}\right]\left[\begin{array}{c} \delta_{y1}\\ \alpha_{1} \end{array}\right]+f_{x1}\left[\begin{array}{cc} p_{11} & p_{12}\\ p_{21} & p_{22} \end{array}\right]\left[\begin{array}{c} \delta_{y1}\\ \alpha_{1} \end{array}\right]+f_{x1}^{2}\left[\begin{array}{cc} q_{11} & q_{12}\\ q_{21} & q_{22} \end{array}\right]\left[\begin{array}{c} \delta_{y1}\\ \alpha_{1} \end{array}\right].$$

(5) $$\delta_{x1}=\frac{t_{1}^{2}f_{x1}}{12L_{1}^{2}}-\frac{1}{2}\left[\begin{array}{cc} \delta_{y1} & \alpha_{1} \end{array}\right]\left[\begin{array}{cc} u_{11} & u_{12}\\ u_{21} & u_{22} \end{array}\right]\left[\begin{array}{c} \delta_{y1}\\ \alpha_{1} \end{array}\right]-f_{x1}\left[\begin{array}{cc} \delta_{y1} & \alpha_{1} \end{array}\right]\left[\begin{array}{cc} v_{11} & v_{12}\\ v_{21} & v_{22} \end{array}\right]\left[\begin{array}{c} \delta_{y1}\\ \alpha_{1} \end{array}\right].$$

(6) $$\left[\begin{array}{c} f_{y2}\\ m_{2} \end{array}\right]=\left[\begin{array}{cc} g_{11} & g_{12}\\ g_{21} & g_{22} \end{array}\right]\left[\begin{array}{c} \delta_{y2}\\ \alpha_{2} \end{array}\right]+f_{x2}\left[\begin{array}{cc} p_{11} & p_{12}\\ p_{21} & p_{22} \end{array}\right]\left[\begin{array}{c} \delta_{y2}\\ \alpha_{2} \end{array}\right]+f_{x2}^{2}\left[\begin{array}{cc} q_{11} & q_{12}\\ q_{21} & q_{22} \end{array}\right]\left[\begin{array}{c} \delta_{y2}\\ \alpha_{2} \end{array}\right].$$

(7) $$\delta_{x2}=\frac{t_{2}^{2}f_{x2}}{12L_{2}^{2}}-\frac{1}{2}\left[\begin{array}{cc} \delta_{y2} & \alpha_{2} \end{array}\right]\left[\begin{array}{cc} u_{11} & u_{12}\\ u_{21} & u_{22} \end{array}\right]\left[\begin{array}{c} \delta_{y2}\\ \alpha_{2} \end{array}\right]-f_{x2}\left[\begin{array}{cc} \delta_{y2} & \alpha_{2} \end{array}\right]\left[\begin{array}{cc} v_{11} & v_{12}\\ v_{21} & v_{22} \end{array}\right]\left[\begin{array}{c} \delta_{y2}\\ \alpha_{2} \end{array}\right].$$

The beam characteristic coefficients may take the BCM coefficients given in Table 1.

The loop closure equations are

(8) $$\alpha_{1}=\alpha_{2}=\alpha_{3}$$

and

(9) $$\left[\begin{array}{c} L_{X}\\ L_{Y}-\Delta_{i} \end{array}\right]=\left[\begin{array}{cc} \cos\theta_{1} & -\sin\theta_{1}\\ \sin\theta_{1} & \cos\theta_{1} \end{array}\right]\left[\begin{array}{c} L_{1}+\Delta_{x1}\\ \Delta_{y1} \end{array}\right]+L_{3}\left[\begin{array}{c} \cos(\theta_{3}+\alpha_{3})\\ \sin(\theta_{3}+\alpha_{3}) \end{array}\right]\left[\begin{array}{cc} \cos(\theta_{2}) & -\sin(\theta_{2})\\ \sin(\theta_{2}) & \cos(\theta_{2}) \end{array}\right]\left[\begin{array}{c} L_{2}+\Delta_{x2}\\ \Delta_{y2} \end{array}\right].$$

where

(10) $$L_{X}=L_{1}\cos\theta_{1}+L_{3}\cos\theta_{3}+L_{2}\cos\theta_{2}$$

and

(11) $$L_{Y}=L_{1}\sin\theta_{1}+L_{3}\sin\theta_{3}+L_{2}\sin\theta_{2}$$

The static balancing equations for $L_{3}$ (for moments, counterclockwise indicates positive):

(12) $$\left[F_{x1}^{\prime }\cos\theta_{1}-F_{y1}^{\prime }\sin\theta_{1}\right]+\left[F_{x2}^{\prime }\cos\left(\pi +\theta_{2}\right)-F_{y2}^{\prime }\sin\left(\pi +\theta_{2}\right)\right]=0$$

(13) $$\left[F_{x1}^{\prime }\sin\theta_{1}+F_{y1}^{\prime }\cos\theta_{1}\right]+\left[F_{x2}^{\prime }\sin\left(\pi +\theta_{2}\right)+F_{y2}^{\prime }\cos\left(\pi +\theta_{2}\right)\right]=0$$

(14) $$M_{1}^{\prime }+M_{2}^{\prime }-F_{y1}^{\prime }L_{3}\cos\left(\theta_{3}-\theta_{1}+\alpha_{3}\right)+F_{x1}^{\prime }L_{3}\sin\left(\theta_{3}-\theta_{1}+\alpha_{3}\right)=0$$

in which

(15) $$\begin{array}{cc} M_{1}^{\prime } & =-M_{1}^{\prime },\;F_{x1}^{\prime }=-F_{x1}^{\prime },\;F_{y1}^{\prime }=-F_{y1}^{\prime },\\ M_{2}^{\prime } & =-M_{2}^{\prime },\;F_{y2}^{\prime }=-F_{y2}^{\prime },\;F_{x2}^{\prime }=-F_{x2}^{\prime }. \end{array}$$

By applying static balancing to $L_{2}$, the force required to displace the bistable element is expressed as

(16) $$F_{i}=F_{x2}\sin\left(\pi +\theta_{2}\right)+F_{y2}\cos\left(\pi +\theta_{2}\right)$$

### 3.2. Simulation Verification

The effectiveness of the BCM in predicting the bistable behavior of the BBCM was validated. The geometric parameters of the mechanism for simulation are summarized in Table 2. The material used for the mechanism is aluminum alloy 7075, with a Young’s modulus of 71.7 GPa.

The finite element simulation was carried out using the Static Structural module in ANSYS Workbench 2022R1. The mounting ends of the BBCM were fixed to reproduce the actual installation condition, and a prescribed displacement was applied to the shuttle along the focusing direction. Large deformation was enabled to capture the geometric nonlinearity and snap-through behavior of the compliant beams. The displacement loading range was set from 0 mm to 2.5 mm with a displacement step of 0.05 mm. At each load step, the reaction force along the focusing direction was extracted from the displacement loading boundary to obtain the force–displacement response curve. The mesh was generated using a surface-size control method, and the mesh size in the key thin-walled regions of the bistable beams was set to 0.5 mm. The convergence of each load step was checked according to the default force and displacement convergence criteria in ANSYS Workbench.

**Figure 7:**
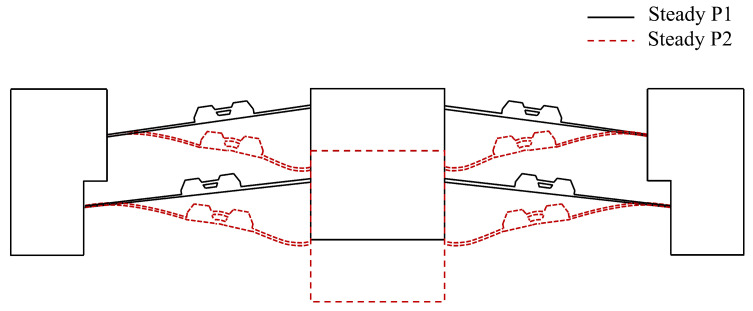
Two steady-state positions of the BBCM.

To validate the BCM, simulation results were obtained using the parameters of the upper beam and lower beam designs, as shown in Table 2. By combining the design parameters of both beams, the performance of the BCM was verified. These parameters were used to simulate the static behavior of the mechanism and assess the accuracy of the BCM’s predictions, with the two steady-state diagrams shown in Figure 7.

**Figure 8:**
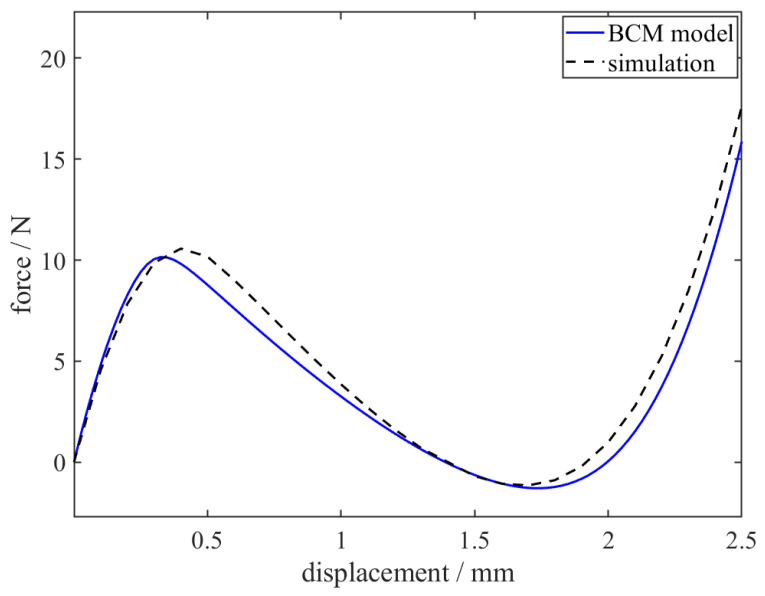
Static behaviors of the BBCM.

As shown in Figure 7 and Figure 8, the BCM captures the overall bistable behavior of the BBCM, including the first peak, the zero-force point, and the local minimum in the negative-stiffness region. To present the comparison more clearly, the key characteristic points of the stiffness response are summarized in Table 3.

The results show that the BCM agrees well with the finite element results in terms of the main feature-point locations, while some discrepancies remain in the force magnitude and in the post-buckling transition region. These differences are mainly related to the simplified assumptions of the BCM. In the BCM, the flexible beams are treated as ideal beam elements, and the effects of shear deformation, local stress redistribution, and deformation coupling in the thin-walled regions are not fully considered. These neglected local effects may accelerate the stiffness variation near the critical region, causing the peak force to appear earlier and leading to deviations in the predicted force magnitude.

## 4. Design of a Flying Strategy and VBM Algorithm

During the flying-shot visual calibration process, the camera remains in continuous motion. Due to shallow DoF optics for high-precision calibration, the imaging system is highly sensitive to substrate warpage and environmental vibrations. Consequently, the target may deviate from the predefined focal plane, making a single focusing operation insufficient to ensure a fully in-focus image.

To address this issue, a combined strategy integrating bistable flying-shot focusing and multi-focus image fusion is adopted. First, a dual-focal-plane acquisition scheme is implemented through the BBCM to capture two images under different focus conditions during motion. Then, an MF image fusion algorithm reconstructs an all-in-focus image, improving robustness against defocus blur and enabling reliable extraction of target features in subsequent processing.

### 4.1. Bistable Flying Strategy

**Figure 9:**
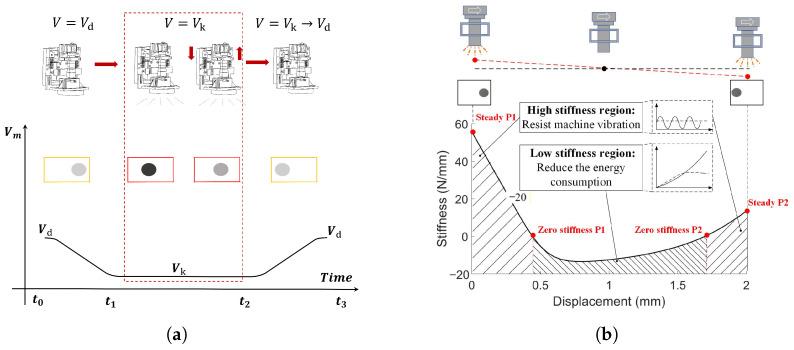
Design of the bistable flying strategy using variable-stiffness characteristics of the BBCM. (**a**) Schematic of the bistable flying strategy. (**b**) Principle of the flying VBM process.

To enable high-efficiency image acquisition under continuous-motion conditions, a bistable flying-shot focusing strategy is further designed, as shown in Figure 9a. Different from the conventional stop-and-capture mode, the camera remains in motion throughout the acquisition process. When the target enters the predefined imaging window, the focusing mechanism rapidly switches between two preset focal planes and captures two images corresponding to different defocus states. These two images are then fused by the GIF-based MF image fusion algorithm. Furthermore, as illustrated in Figure 9b, this strategy leverages the variable-stiffness characteristics of the BBCM. The high-stiffness regions at the two steady states ensure imaging stability against machine vibration, while the low-stiffness region in the middle facilitates rapid and energy-efficient focal-plane transitions.

Under flying-shot conditions, platform velocity is first constrained by motion blur. Let *W* denote the field-of-view width, let the image width contain 2448 pixels, and let *t* denote the exposure time. To ensure that the image blur caused by a single exposure does not exceed one pixel, the allowable flying-shot velocity is defined as:

(17) $$v_{k}=\frac{W}{2448\;t}$$

According to the bistable flying-shot focusing strategy, the motion platform runs at the normal velocity $v_{d}$ outside the acquisition region and decreases to the allowable velocity $v_{k}$ when entering the dual-focus imaging window. A simple piecewise velocity profile can be expressed as:

(18) $$v_{m}\left(t\right)=\left\{\begin{array}{cc} v_{d}-\frac{v_{d}-v_{k}}{t_{1}-t_{0}}\left(t-t_{0}\right), & t_{0}\leq t<t_{1}\\ v_{k}, & t_{1}\leq t<t_{2}\\ v_{k}+\frac{v_{d}-v_{k}}{t_{3}-t_{2}}\left(t-t_{2}\right), & t_{2}\leq t<t_{3} \end{array}\right.$$

In this way, the target images can be captured within the allowable blur range while maintaining the overall operating efficiency of the LDI machine. By combining the dual-focus acquisition strategy with the adopted guided-filter-based multi-focus fusion method, the system avoids the time-consuming process of point-by-point auto focus search and achieves efficient image acquisition and target reconstruction under continuous-motion conditions.

### 4.2. Guided-Filter-Based Multi-Focus Image Fusion

**Figure 10:**
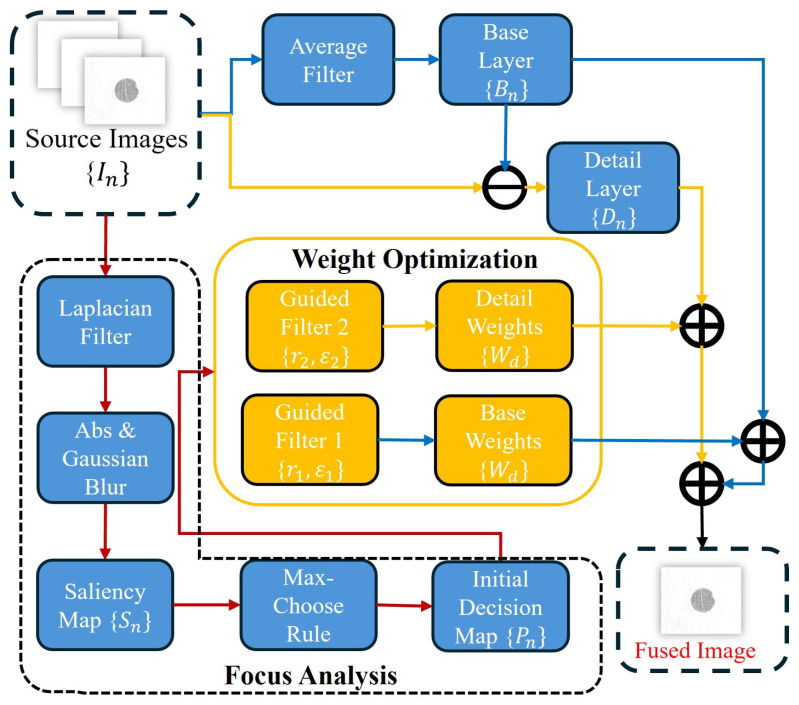
Flowchart of the GIF-based multi-focus image fusion algorithm.

A guided-filter (GIF)-based two-scale MF image fusion method is employed in this work, following the guided-filter image fusion framework reported in [31]. In this study, this method is adopted as an image preprocessing module for the dual-focal-plane acquisition strategy and subsequent target recognition. As illustrated in Figure 10, the fusion framework consists of five main steps: two-scale decomposition, saliency analysis, initial decision-map construction, GIF-based weight optimization, and image reconstruction. The source image sequence is denoted as:

(19) $$\left\{I_{n}\left(x,y\right)|n=2\right\}.$$

Each source image is first decomposed into a base layer and a detail layer through average filtering.

(20) $$B_{n}\left(x,y\right)=I_{n}\left(x,y\right)*Z_{\mathrm{avg}},$$

(21) $$D_{n}\left(x,y\right)=I_{n}\left(x,y\right)-B_{n}\left(x,y\right),$$

where $Z_{\mathrm{avg}}$ denotes the average filter.

To evaluate the sharpness distribution of each source image, a Laplacian operator is used to extract high-frequency information.

(22) $$H_{n}\left(x,y\right)=\left|I_{n}\left(x,y\right)*L\right|,$$

where *L* is the Laplacian kernel. The corresponding saliency map is then obtained by Gaussian smoothing.

(23) $$S_{n}\left(x,y\right)=H_{n}\left(x,y\right)*g_{r_{g},\sigma_{g}},$$

where $g_{r_{g},\sigma_{g}}$ is the Gaussian low-pass filter.

Based on the saliency maps, the initial binary decision map is generated by the winner-take-all rule.

(24) $$P_{n}\left(x,y\right)=\left\{\begin{array}{cc} 1, & S_{n}\left(x,y\right)=\max\left(S_{1}\left(x,y\right),S_{2}\left(x,y\right),\ldots ,S_{N}\left(x,y\right)\right),\\ 0, & \mathrm{otherwise}. \end{array}\right.$$

To improve spatial consistency and suppress boundary artifacts, guided filtering is further introduced to optimize the weight maps of the base and detail layers.

(25) $$W_{n}^{B}\left(x,y\right)=G_{r_{1},\varepsilon_{1}}\left(P_{n}\left(x,y\right),I_{n}\left(x,y\right)\right),$$

(26) $$W_{n}^{D}\left(x,y\right)=G_{r_{2},\varepsilon_{2}}\left(P_{n}\left(x,y\right),I_{n}\left(x,y\right)\right),$$

where $G_{r,\varepsilon }\left(\cdot \right)$ denotes the guided-filtering operator, and $r_{1},\varepsilon_{1}$ and $r_{2},\varepsilon_{2}$ are the GIF parameters for the base and detail layers, respectively.

Since the weight coefficients at the same pixel location should satisfy the summation constraint, the optimized weights are normalized as

(27) $${\bar{W}}_{n}^{B}\left(x,y\right)=\frac{W_{n}^{B}\left(x,y\right)}{\sum_{j=1}^{N}W_{j}^{B}\left(x,y\right)},$$

(28) $${\bar{W}}_{n}^{D}\left(x,y\right)=\frac{W_{n}^{D}\left(x,y\right)}{\sum_{j=1}^{N}W_{j}^{D}\left(x,y\right)}.$$

Finally, the fused image is reconstructed by combining the fused base and detail layers.

(29) $$F\left(x,y\right)=\sum_{n=1}^{N}\left(B_{n}\left(x,y\right)\cdot {\bar{W}}_{n}^{B}\left(x,y\right)+D_{n}\left(x,y\right)\cdot {\bar{W}}_{n}^{D}\left(x,y\right)\right).$$

This method preserves edge and texture information while reducing artifacts, thereby providing a reliable input for subsequent target feature extraction.

### 4.3. RRHT-Based VBM Algorithm

To improve the robustness of circular target recognition under nonuniform illumination, edge degradation, and background interference, this study incorporates an RRHT-based target recognition algorithm into the existing VBM framework. The adopted recognition pipeline follows previous work on LDI target recognition and is further integrated with the proposed dual-focus acquisition scheme and the guided-filter-based multi-focus image fusion method.

**Figure 11:**
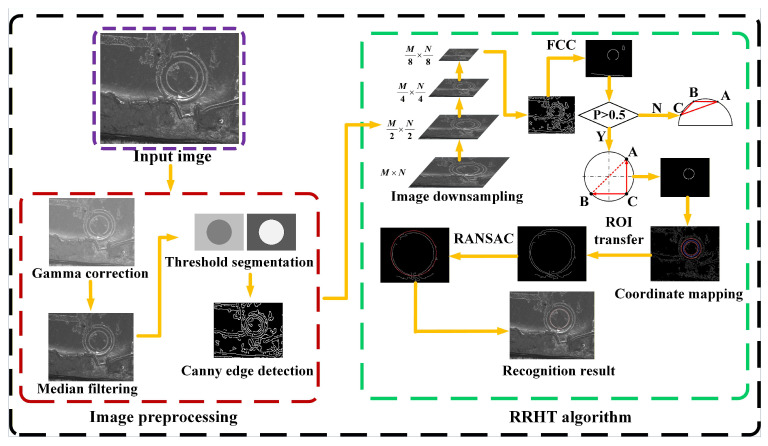
Flowchart of the RRHT-based target recognition algorithm.

As shown in Figure 11, the algorithm mainly consists of two stages, namely, image preprocessing and RRHT-based circular target recognition. In the preprocessing stage, gamma correction and median filtering are first applied to suppress noise and enhance image contrast. Threshold segmentation and Canny edge detection are then used to extract the contour features of the target. After that, the edge image is downsampled to reduce the computational cost while preserving the main geometric features of the circular target for the subsequent recognition process.

The RRHT algorithm determines candidate circles by using an adaptive point-selection strategy. When the contour completeness of the circular target is relatively high, one-point sampling is adopted to improve the sampling efficiency. When the contour is incomplete or degraded, the conventional three-point strategy is retained to avoid invalid fitting.

Based on the preprocessed edge image, the algorithm first generates candidate circles in the downsampled image and then verifies these candidate circles and maps them to the original-resolution image for local refinement. To suppress the effects of pseudo-edges, contour discontinuities, and local outliers, a RANSAC-based refitting procedure is finally performed within the mapped region of interest.

The RRHT-based recognition algorithm finally outputs the circle center coordinates and the fitted radius of the target. These extracted geometric parameters are used in Section 5.5 to evaluate the horizontal error, vertical error, comprehensive target-center error, and radius error under loaded flying-shot conditions.

## 5. Prototype Test of the BBCM

### 5.1. Prototype Fabrication and Test Setup

**Figure 12:**
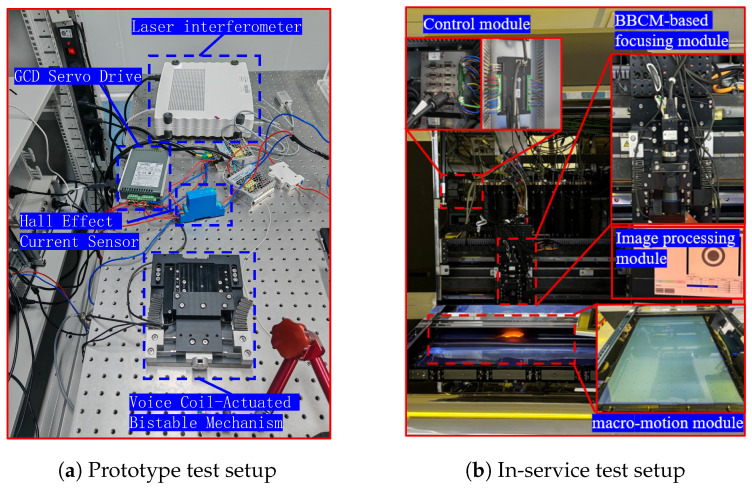
Test setup of the BBCM prototype and in-service integration.

The prototype of the VCM-actuated BBCM is mounted on an optical vibration isolation table to eliminate external disturbances. The VCM is driven by a GCD servo drive, which provides precise motion control commands. A Hall effect current sensor is integrated into the control circuit to monitor the driving current in real time. The collected current data are essential to calculating the equivalent driving force and the input electrical energy consumption. A high-precision laser interferometer is utilized to measure the real-time output displacement of the BBCM. To evaluate the static and dynamic performance of the proposed BBCM, a comprehensive experimental testbed was constructed, as illustrated in Figure 12a. The BBCM is integrated into the LDI machine, and the in-service platform is shown in Figure 12b. This setup ensures the accurate acquisition of the stiffness and energy characteristics during the state-switching process.

A flying focusing VBM system is developed based on this platform. The VBM is divided into five modules: (1) A BBCM-based focusing module mounted on the camera axis, enabling rapid switching between two preset focal planes during continuous flying focusing; (2) an image processing and interactive interface module for target display, image fusion, and experimental data recording; (3) a macro-motion module for carrying and positioning PCB substrates; (4) a UD module responsible for signal interaction and trigger coordination; (5) a control module for motion execution and multi-axis coordination.

Through the collaboration of these modules, the system achieves performance evaluation under flying focusing conditions.

This section focuses on the no-load basic performance verification of the BBCM and the constant-stiffness CM. The core test indicators include stiffness characteristics, stiffness distribution, energy consumption, displacement responses, and tracking errors. The test results are analyzed based on experimental data and corresponding diagrams to verify the rationality of the BBCM design and lay a foundation for subsequent load condition tests.

### 5.2. Stiffness Characteristic Test of the BBCM

**Figure 13:**
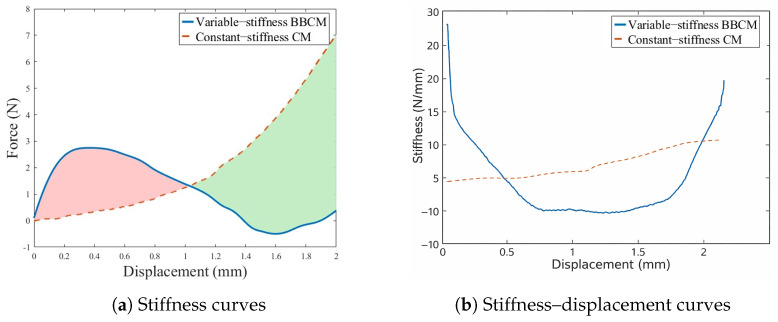
Stiffness characteristic test of the BBCM and constant-stiffness CM. Red indicates the region where the BBCM exhibits higher energy consumption, while green indicates the region where the BBCM exhibits lower energy consumption.

The results of the no-load stiffness test are presented in Figure 13a. The red area indicates where the BBCM consumes more energy than the constant-stiffness CM, while the green area shows where the constant-stiffness CM consumes more energy than the BBCM.

The constant-stiffness compliant mechanism exhibits a monotonically increasing linear force response without obvious stable states, requiring a continuous increase in driving force to overcome elastic resistance.

In contrast, the BBCM demonstrates significant nonlinear bistable characteristics, forming two stable equilibrium positions at 0.4 mm and 1.6 mm. It can be seen that the force decreases to 0 N at 1.4 mm, which is consistent with the bistable “snap-through” effect. That is, a rapid snap-through transition between two stable states can be achieved, and the new state can be maintained without continuous energy input after switching, enabling state transition with a relatively small driving force.

The stiffness comparison results are shown in Figure 13b. The dashed part represents the constant-stiffness compliant mechanism, which maintains stable stiffness throughout the stroke with an average of 5 N/mm, ensuring a certain level of stability but leading to high energy consumption.

In contrast, the BBCM exhibits a dynamic stiffness distribution: the peak stiffness at the stable positions is 35 N/mm, which is 51.5% higher than that of the constant-stiffness compliant mechanism, capable of resisting micro-vibrations to ensure static stability. Within the transition zone, the stiffness decreases significantly and presents a negative stiffness of −10 N/mm, reducing the driving force required for state switching.

It can be seen that the green energy-saving region occupies the main part of the focusing stroke, and the driving force required for state switching is reduced in comparison with rigid mechanisms, which provides support for the subsequent verification of the energy-saving effect of the BBCM.

### 5.3. Energy-Consumption Test of the BBCM

**Figure 14:**
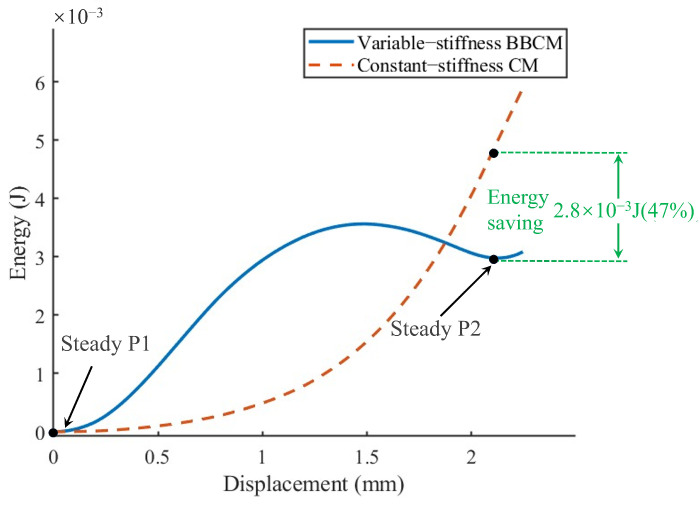
Cumulative mechanical energy consumption of the BBCM and constant-stiffness CM.

To further evaluate the energy-saving effect of the proposed bistable mechanism, the cumulative mechanical energy consumption of the bistable mechanism and the constant-stiffness mechanism over the full focusing stroke was compared, as shown in Figure 14. When moving from the first stable state to the second stable state, the mechanical energy consumption of the bistable mechanism was $3.10\times 10^{-3}\;J$, whereas that of the constant-stiffness mechanism was $5.90\times 10^{-3}\;J$.

As illustrated in Figure 14, the cumulative energy curve of the bistable mechanism rises rapidly in the initial displacement interval, reaches a peak at approximately 1.46mm, and then decreases slightly before remaining at a relatively low level near the end of the stroke. In contrast, the cumulative energy of the constant-stiffness mechanism increases continuously with displacement and becomes significantly higher than that of the bistable mechanism in the later stage of the stroke. It can be observed that over the complete displacement range, the final cumulative mechanical energy consumption of the bistable mechanism is markedly lower than that of the constant-stiffness mechanism. Although the bistable mechanism needs to overcome a local energy barrier during the initial stage of switching, its overall cumulative mechanical energy consumption after stable-state transition remains lower than that of the constant-stiffness mechanism.

Experiments verify that the proposed bistable mechanism can maintain high local stiffness near its stable positions while reducing the total energy requirement during the entire switching process, thus providing support for continuous operation under flying focusing conditions.

Table 4 summarizes the key force, stiffness, and energy characteristics of the BBCM and the constant-stiffness CM. The results indicate that the proposed BBCM exhibits a distinct peak-force characteristic, higher local stiffness near the working positions, and lower cumulative mechanical energy over the full stroke.

### 5.4. Step Response Test of the BBCM

**Figure 15:**
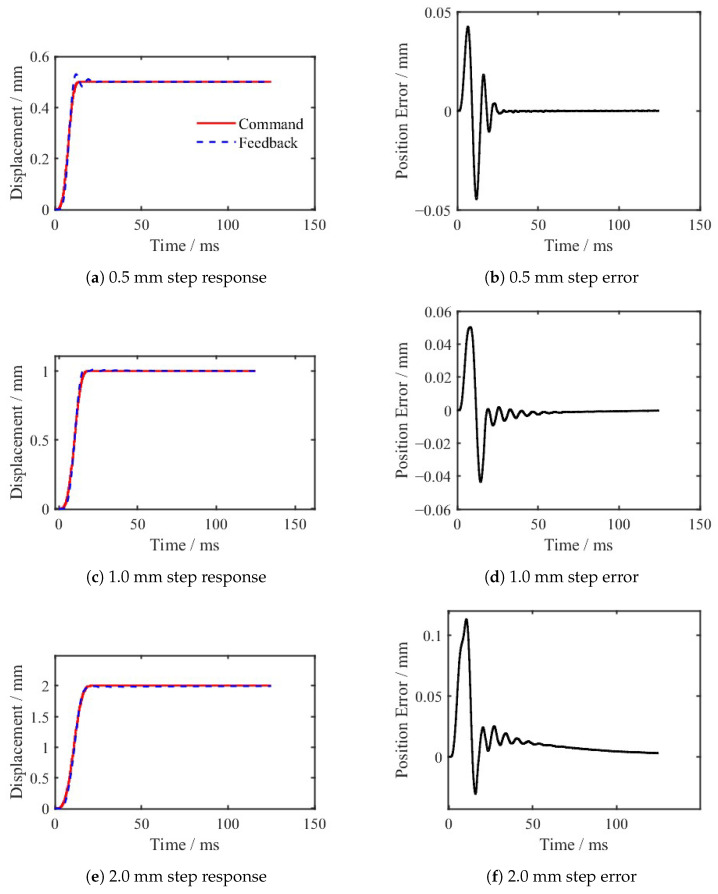
Step response curves and step error curves under different command amplitudes.

Step tests with amplitudes of 0.5 mm, 1.0 mm, and 2.0 mm were performed on the BBCM prototype. The displacement responses and tracking errors for each case were recorded, as illustrated in Figure 15.

The motion control parameters were adjusted for various step command amplitudes to achieve an appropriate balance between response speed and stability. Meanwhile, the overshoots of the BBCM under different command amplitudes during step response were obtained and are summarized in Table 5.

Step response curves show that the BBCM achieves stable and oscillation-free step tracking under step inputs of 0.5 mm, 1.0 mm, and 2.0 mm. At the small stroke of 0.5 mm, the response is the fastest (rise time of 4.75 ms), but the overshoot reaches 6.00%. Under the 1.0 mm condition, the overshoot drops to 1.03% and the settling time is 11.44 ms, achieving a good balance between speed and stability.

For the large stroke of 2.0 mm, the overshoot is only 0.01%, and although the rise time is extended to 8.38 ms and the steady-state error increases slightly, the overall error remains at the micrometer level. The error curves show that the 0.5 mm has the optimal steady-state accuracy (steady-state error of −0.05 μm and steady-state root-mean-square error of 0.08 μm).

For 2.0 mm, the error and steady-state fluctuation increase slightly, but still meet the requirements of high-precision positioning. Experiments verify that the proposed BBCM exhibits favorable dynamic tracking performance and satisfactory positioning accuracy, making it suitable for fast-focusing applications and providing support for subsequent in-service performance tests.

### 5.5. In-Service Performance Test Inside the LDI Machine

Two types of verification experiments were implemented. First, the target recognition errors of the BBCM and the constant-stiffness CM were compared under the loaded flying-shot condition to evaluate whether the BBCM-based focusing unit can maintain comparable visual measurement performance. Second, the cumulative electrical and mechanical energy consumption of the two mechanisms were compared under the same loaded flying-shot condition to evaluate the energy-saving capability of the BBCM.

To verify the effectiveness of the proposed mechanism in practical visual calibration, the target recognition performance of the BBCM is compared with that of the constant-stiffness CM under loaded flying-shot conditions. The positioning accuracy is evaluated by comparing the target-center coordinates and fitted radius obtained under flying-shot focusing with the reference values extracted from accurately focused fixed-point images. To quantitatively evaluate the target-center extraction accuracy, the horizontal error $e_{x}$, vertical error $e_{y}$, and comprehensive target-center error $e_{p}$ are defined as

(30) $$e_{x}=\left|x-x_{0}\right|,\;e_{y}=\left|y-y_{0}\right|,\;e_{r}=\left|r-r_{0}\right|$$

(31) $$e_{p}=\sqrt{e_{x}^{2}+e_{y}^{2}}$$

where (x,y) and *r* denote the target-center coordinates and fitted radius obtained under flying-shot focusing, while $\left(x_{0},y_{0}\right)$ and $r_{0}$ denote the reference values extracted from accurately focused fixed-point images.

**Figure 16:**
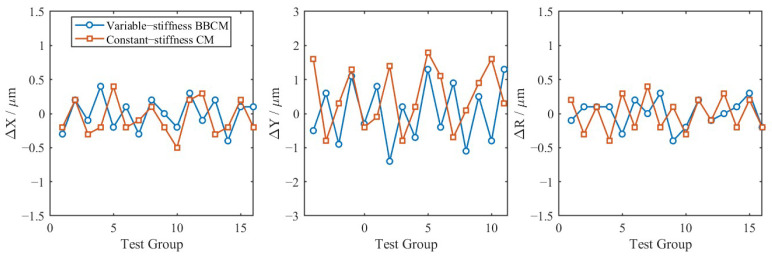
Comparison of target recognition errors between the BBCM and the constant-stiffness CM.

The target recognition errors in the horizontal coordinate, vertical coordinate, and fitted radius are presented in Figure 16. The two mechanisms show similar error levels in the horizontal and radial directions, while a clearer difference appears in the vertical direction. The BBCM shows comparable fluctuation levels to the constant-stiffness CM.

**Figure 17:**
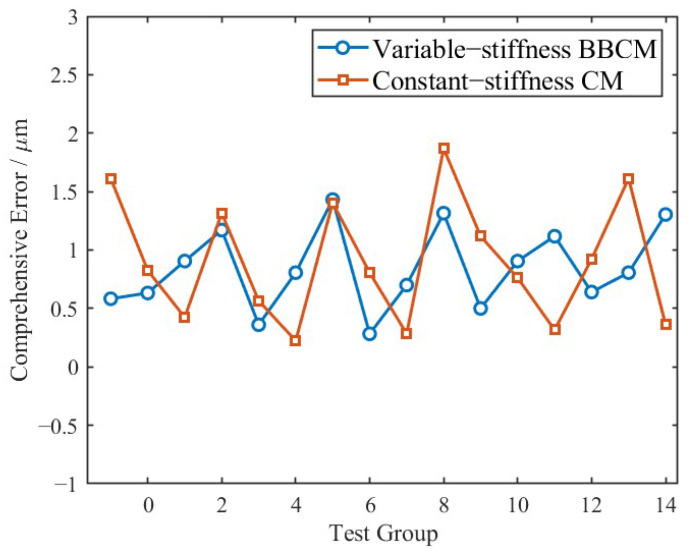
Comparison of comprehensive target-center error between the BBCM and the constant-stiffness CM.

The comprehensive target-center error over 16 test groups is shown in Figure 17. The two mechanisms show close error levels, while the BBCM exhibits a slightly lower average comprehensive error under the tested condition.

The average recognition errors of the two mechanisms are summarized in Table 6. The average comprehensive center error of the BBCM is 0.88 μm, whereas that of the constant-stiffness mechanism is 0.90 μm. This corresponds to a reduction of 2.22% in the overall target-center recognition error.

The average vertical coordinate error decreases from 0.84 μm to 0.80 μm, while the average comprehensive target-center error decreases from 0.90 μm to 0.88 μm. Although the average horizontal coordinate error and radius error of the two mechanisms remain close, the BBCM shows a slightly lower comprehensive error under the tested loaded flying-shot condition. As shown in Table 7, the standard deviations of the *x*-coordinate, *y*-coordinate, and fitted radius of the BBCM are close to or slightly smaller than those of the constant-stiffness CM. Therefore, the recognition result only indicates a slight improvement tendency and should not be regarded as a generalized conclusion.

The target recognition comparison is mainly used to verify that the BBCM-based focusing unit can maintain comparable visual measurement performance under camera-loaded flying-shot conditions. Since the difference in recognition error is small, the visual recognition result is not used as the primary evidence for performance improvement. The main advantage of the proposed BBCM is further reflected by the energy-consumption comparison, where the bistable stiffness regulation effectively reduces the driving energy required for focal-plane switching.

**Figure 18:**
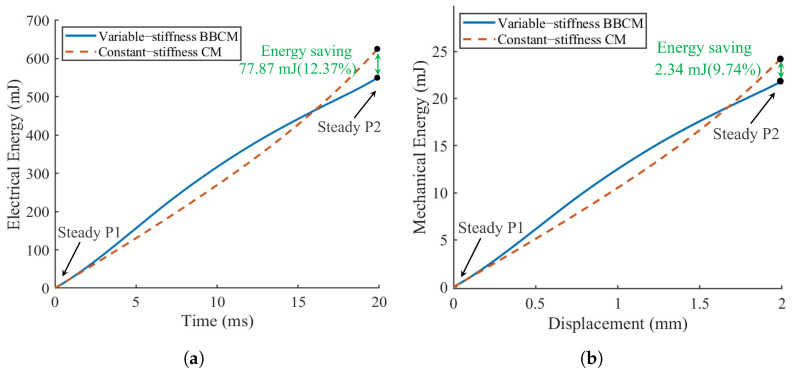
Comparison of average cumulative focusing energy consumption between the BBCM and the constant-stiffness CM under camera-loaded flying-shot operation. (**a**) Average electrical energy curves. (**b**) Average mechanical energy curves.

To further assess the practical energy-saving capability of the proposed mechanism, the average cumulative focusing energy consumption of the BBCM and the constant-stiffness CM was compared under camera-loaded flying-shot operation, as shown in Figure 18. The electrical energy and mechanical energy were evaluated separately to characterize the input energy consumption and the mechanical work during the focusing process. The input electrical energy was calculated by integrating the product of the bus voltage and the measured motor current over time:

(32) $$E_{\mathrm{elec}}\left(t\right)=\int_{0}^{t}V_{\mathrm{bus}}\left|I(\tau )\right|\;d\tau .$$

where $E_{\mathrm{elec}}\left(t\right)$ is the cumulative input electrical energy, $V_{\mathrm{bus}}$ is the bus voltage, I(τ) is the measured motor current, and *t* is the focusing time. The absolute value of the current is used to represent the consumed electrical energy during the focusing process.

The mechanical energy was calculated by integrating the force–displacement response:

(33) $$E_{\mathrm{mech}}\left(x\right)=\int_{0}^{x}F\left(\xi \right)\;d\xi .$$

where $E_{\mathrm{mech}}\left(x\right)$ is the cumulative mechanical energy, F(ξ) is the driving force along the focusing direction, ξ is the displacement integration variable, and *x* is the focusing displacement. In the calculation, 1Nmm=1mJ.

The energy-consumption test of the BBCM was repeated three times under the same camera-loaded flying-shot condition. The focusing time of the BBCM was approximately 20 ms during the focal-plane switching process. As shown in Figure 18, the average cumulative energy curves were used to compare the energy-consumption characteristics of the BBCM and the constant-stiffness CM. Table 8 summarizes the repeatability of the BBCM energy-consumption measurements at 2 mm. The final electrical and mechanical energy values are reported as means ± standard deviations, and the coefficient of variation was used to evaluate the repeatability of the measurements.

The energy-consumption test of the BBCM was repeated three times under the same camera-loaded flying-shot condition. The experimentally measured focusing time of the BBCM was approximately 20 ms during the focal-plane switching process. Table 8 summarizes the repeatability of the BBCM energy-consumption measurements at 2 mm. The final electrical and mechanical energy values are reported as means ± standard deviations, and the coefficient of variation was used to evaluate the repeatability of the measurements.

As shown in Figure 18, at the end of the focusing process, the cumulative energy consumption of the BBCM is lower than that of the constant-stiffness CM. Specifically, the electrical energy consumption is reduced by 77.87 mJ, corresponding to a reduction of 12.37%, while the mechanical energy consumption is reduced by 2.34 mJ, corresponding to a reduction of 9.74%. This result indicates that bistable stiffness regulation is beneficial for reducing the driving energy required for focal-plane switching.

Overall, the camera-loaded flying-shot experiments show that the proposed BBCM can maintain comparable visual measurement performance while reducing focusing energy consumption. Therefore, the main advantage of the proposed mechanism lies in its energy-saving capability during dual-position focal-plane switching.

## 6. Conclusions

This study presents a VCM-driven BBCM for the flying-shot visual calibration system of an LDI machine. By introducing a ligament-inspired variable-stiffness design, the proposed mechanism achieves low equivalent stiffness during focal-plane transition and relatively high local stiffness near the two stable positions. The theoretical model and finite element analysis show consistent bistable characteristics, and the prototype experiments further confirm the feasibility of the proposed BBCM design. The main contribution of this work lies in the mechanism-level design, modeling, and experimental validation of the BBCM for energy-efficient dual-position focusing in LDI flying-shot VBM systems.

The no-load experiments show that the proposed BBCM exhibits a clear bistable stiffness response, reduced cumulative mechanical energy, and favorable dynamic response characteristics. Compared with the constant-stiffness compliant mechanism, the BBCM maintains higher local stiffness near the working positions while reducing the energy demand during focal-plane switching. In addition, step-response tests under command amplitudes of 0.5 mm, 1.0 mm, and 2.0 mm verify that the mechanism can achieve millisecond-level focusing motion, indicating its applicability to fast-focusing tasks.

The loaded flying-shot experiments further verify the practical feasibility of the proposed system. The guided-filter-based multi-focus image fusion method and the RRHT-based target recognition method are adopted as supporting tools for visual performance evaluation, rather than as independent algorithmic innovations of this paper. Under the tested camera-loaded condition, the BBCM-based system shows comparable target recognition repeatability to the constant-stiffness CM. The average comprehensive target-center error decreases slightly from 0.90 μm to 0.88 μm. Considering the small improvement magnitude, this result is interpreted as a slight improvement tendency rather than a generalized conclusion on recognition stability. In contrast, the energy-consumption comparison provides clearer evidence for the advantage of the BBCM. The electrical energy consumption is reduced by 77.87 mJ, corresponding to a reduction of 12.37%, while the mechanical energy consumption is reduced by 2.34 mJ, corresponding to a reduction of 9.74%. These results indicate that bistable stiffness regulation is beneficial for reducing the driving energy required for focal-plane switching.

There are still several limitations in the present work. First, the loaded flying-shot visual tests were conducted under a limited number of experimental conditions, and the observed recognition improvement remains small. Therefore, larger-sample statistical validation is required in future work. Second, the energy-consumption tests were mainly carried out under the current camera-loaded condition, and additional repeated tests under different flying-shot speeds, loads, and disturbance levels are needed to further evaluate the robustness of the energy-saving effect. Third, the long-term fatigue behavior and repeatability retention of the BBCM after high-cycle switching have not yet been fully investigated. Future work will focus on long-duration reliability tests, improved control strategies, and broader validation of the BBCM-based focusing system in practical LDI production scenarios.

## Figures and Tables

**Table 1:** Beam characteristic coefficients for the BCM.

$g_{11}$	$g_{12}=g_{21}$	$g_{22}$	$p_{11}=u_{11}$	$p_{12}=p_{21}$ $u_{12}=u_{21}$	$p_{22}=u_{22}$	$q_{11}=v_{11}$	$q_{12}=q_{21}$ $v_{12}=v_{21}$	$q_{22}=v_{22}$
12	−6	4	6/5	−1/10	2/15	−1/700	1/1400	−11/6300

**Table 2:** Design parameters of the BBCM.

Parameter	Upper Beam	Lower Beam
*W*/mm	15	15
$L_{1}$/mm	14.5	20.5
$\theta_{1}$/°	88.3	87.8
$t_{1}$/mm	0.33	0.37
$L_{2}$/mm	10	12
$\theta_{2}$/°	87.7	87.7
$t_{2}$/mm	0.33	0.37
$L_{3}$/mm	10.5	12.5
$\theta_{3}$/°	88.3	87.8

**Table 3:** Comparison of key feature points between the BCM and finite element results.

Characteristic Parameter	BCM	Finite Element Result
First peak displacement, mm	0.325	0.400
First peak force, N	10.153	10.572
First zero-force–displacement, mm	1.390	1.400
Displacement at minimum negative force, mm	1.725	1.700
Minimum negative force, N	−1.293	−1.156

**Table 4:** Comparison of key characteristics between the BBCM and constant-stiffness CM.

Index	BBCM	Constant-Stiffness CM
Peak force (N)	2.75	—
Initial stiffness (N/mm)	26.0	4.5
Final stiffness (N/mm)	19.5	10.5
Mechanical energy (J)	$3.10\times 10^{-3}$	$5.90\times 10^{-3}$

**Table 5:** Parameter settings and overshoot under different step amplitudes.

Step Amplitude (mm)	Speed (mm/s)	Acceleration (mm/s^2^)	Velocity Feedforward	Position Gain	Overshoot (%)
0.5	300	20,000	0.95	850	6.00
1.0	300	20,000	0.90	800	1.03
2.0	300	25,000	0.80	700	0.01

**Table 6:** Comparison of target recognition errors under different focusing mechanisms.

Mechanism	Centroid *x*-Error, μm	Centroid *y*-Error, μm	Comprehensive Error, μm	Radius Error, μm
Variable-stiffness BBCM	0.25	0.80	0.88	0.25
Constant-stiffness CM	0.24	0.84	0.90	0.23

**Table 7:** Repeatability statistics of target recognition results under loaded flying-shot conditions.

Metric	BBCM	Constant-Stiffness CM
Standard deviation of *x*-coordinate, μm	0.237	0.258
Standard deviation of *y*-coordinate, μm	0.905	0.909
Standard deviation of fitted radius *r*, μm	0.208	0.257

**Table 8:** Repeatability of BBCM energy-consumption measurements at 2 mm under camera-loaded flying-shot conditions.

Energy Type	Final Energy, mJ	CV, %
Electrical energy	536.777±0.521	0.097
Mechanical energy	21.852±0.022	0.101

## Data Availability

The datasets used and/or analyzed during the current study are available from the corresponding author upon reasonable request.

## Abbreviations

The following abbreviations are used in this manuscript:

IC	Integrated circuit
HDI	High-density interconnect
VBM	Vision-based measurement
LDI	Laser-direct imaging
PCB	Printed circuit board
CM	Compliant mechanism
BCM	Beam constraint model
BBCM	Bionic bistable compliant mechanism
DoF	Depth of field
VCM	Voice coil motor
RRHT	Revised randomized Hough transformation
